# Pet-related bacterial zoonotic infections: Three cases of severe infections in the immunocompromised host

**DOI:** 10.1016/j.idcr.2022.e01623

**Published:** 2022-09-26

**Authors:** E.M. van Wezel, E.S.J. van der Beek, M.A.N. Siebrecht, A.J. Stel, M. Wouthuyzen-Bakker, N.E.L. Meessen

**Affiliations:** a Department of Medical Microbiology and Infection Prevention, University of Groningen, University Medical Center Groningen, Groningen, the Netherlands; b Department of Plastic surgery, University of Groningen, University Medical Center Groningen, Groningen, the Netherlands; c Department of Rehabilitation Medicine, University of Groningen, University Medical Center Groningen, Groningen, the Netherlands; d Department of Rheumatology, University of Groningen, University Medical Center Groningen, Groningen, the Netherlands

**Keywords:** Zoonosis, Pet-related infection, *Helicobacter canis*, *Pasteurella multocida*, *Capnocytophaga canimorsus*

## Abstract

Pets can have many positive effects on their owners. However, close contact with pets offers optimal conditions for transmission of micro-organisms. Especially immunocompromised patients are at risk for zoonotic infections. Here we describe the diagnosis, microbiology and treatment of three patients with severe zoonotic infections with *Helicobacter canis, Pasteurella multocida and Capnocytophaga canimorsus*. With this case report we would like to emphasize the importance of awareness for pet-related zoonotic infections in immunocompromised patients.

## Introduction

More than 80 million European households are estimated to own at least one pet. Pets can have numerous beneficial psychological and physical effects on their owners [1]. However, close contact between humans and domestic animals offers optimal conditions for transmission of micro-organisms. It has been implicated that pets can transmit a zoonosis to their owners, especially in immunocompromised patients [2]. Many different routes of bacterial transmission have been described, with bites and hand-to mouth contact being the most common routes [2], [3].

The two most common bacteria transmitted from pets to humans are *Campylobacter* and *Salmonella* species, causing zoonotic gastroenteritis [4], [5]. In skin infections following dog bites and cat scratches *Pasteurella spp* are the most frequently isolated micro-organisms.

Here, we describe three immunocompromised patients with severe pet-related bacterial zoonotic infections.

### Case description

*Patient 1*, A 62 year old male with a history of type II diabetes, deep venous thrombosis and stage IV peripheral arterial disease presented to the Emergency Department because of pain and swelling of the left hand. The patient had small abrasions on the hand. Laboratory examination revealed an elevated C-reactive protein ( 93 mg/L) and a leukocytosis (12,1 × 10^9^/L). The swelling progressed to a panaritium tendineum and patient was treated with surgical incision, drainage and intravenous flucloxacillin.

*Microbiology* Two days after presentation, *Pasteurella multocida* was cultured from the wound on blood agar with 5% sheep blood (BA) and chocolate agar (CHOC). Several days later *Bacteroides pyogenes* was cultured in the anaerobic culture. Antimicrobial susceptibility of the *Pasteurella multocida* was performed by disk diffusion on Mueller-Hinton agar + 5% defibrinated horse blood and 20 mg/L ß-NAD as advised by Eucast. The *Pasteurella multocida* was reported susceptible to amoxicillin**.** The *Bacteroides pyogenes* was reported susceptible to amoxicillin, amoxicillin/clavulanic acid, clindamycin and metronidazole.

*Clinical course* 2 days after admission repeated surgery was performed, however 4 days after admission partial amputation of digit IV of the left hand was necessary. Once culture results became available antimicrobial therapy was switched to intravenous amoxicillin/clavulanic acid 1000/200 mg 4 times a day. Because no clinical improvement was observed and susceptibility results of the *Bacteroides* were not available yet, metronidazole was added to the amoxicillin/clavulanic acid therapy. Despite this treatment a third surgical drainage was performed a few days later. After this the patient improved and the intravenous antibiotics were switched to oral amoxicillin/clavulanic acid and metronidazole for a total duration of 3 weeks.

Upon inquiry, the patient turned out to own a cat. The cat probably licked abrasions on the hand of the patient, which may have caused the infection.

*Patient 2*, a 49-year-old female with a history of rheumatoid arthritis, for which she was treated with immunosuppressive drugs, presented to the Emergency department with a 2 week history of pain, swelling and erythema of the left ankle and calf. She also suffered from malaise and nausea. Upon presentation the patient was hemodynamically stable and had no fever. Laboratory examination revealed an elevated C-reactive protein (189 mg/L) and a leukocytosis (11,1 ×10^9^/L). The patient was admitted and empirically treated for cellulitis with intravenous amoxicillin/clavulanic acid 1000/200 mg 4 times a day.

**Fig. 1:**
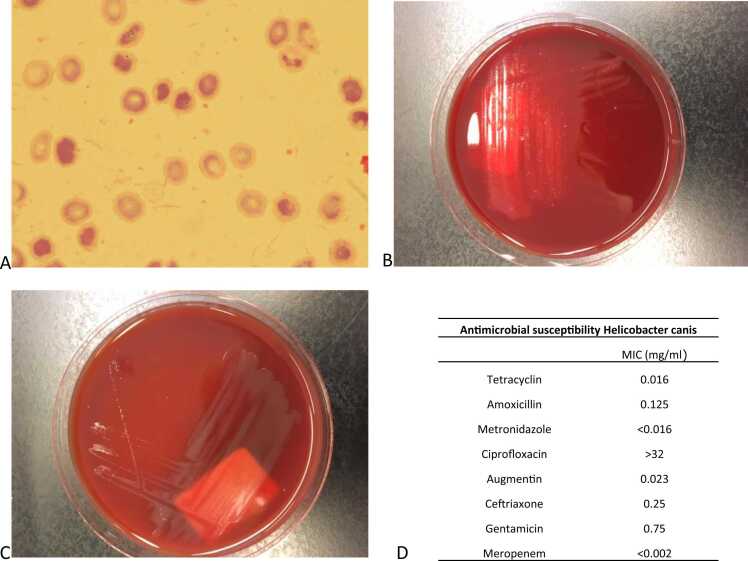
a. Gram stain of the blood culture bottle from patient 2 showing curved Gram-negative rods. **b.** Small golden colonies on BHM agar. **c**. Growth on BBA agar. **d.** Antimicrobial susceptibility results. MICs for taetracyclin, amoxicillin, metronidazole, ciprofloxacin, amoxicillin/calvulanic acis, ceftriaxone, gentamicine and meropenem are shown.

*Microbiology* After 7 days of incubation 1 out of 2 blood culture bottles (aerobic bottle) was reported positive. Gram stain revealed the presence of curved Gram-negative rods (Fig. 1A). The bottle was sub-cultured onto BA and CHOC under aerobic conditions and Brucella blood agar + 5% sheep blood (BBA), belo horizonte medium (BHM), Campylobacter selective agar and Campylobacter blood free selective agar under micro-aerophilic conditions (6% O2 and 7,2% CO2) at 35Cº. Bacterial growth was observed on the BBA and BHM plates (small golden colonies) (Fig. 1B and C).

**Fig. 2:**
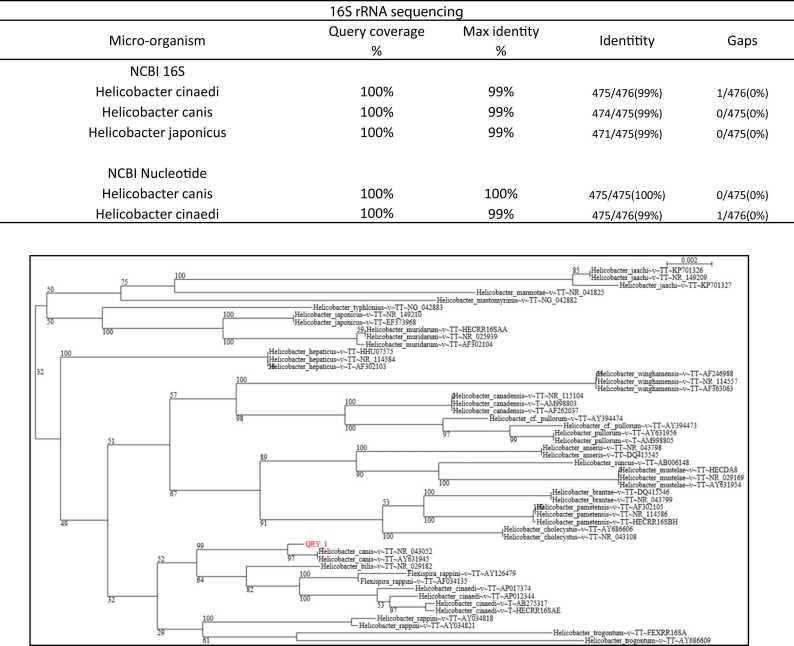
16 S rRNA sequencing results from the gram negative curved rod in the blood culture of patient 2.

Matrix Assisted Laser Desorption/Ionization Time Of Flight Mass Spectrometry (MALDITOF MS, Bruker Microflex) identified *Helicobacter canis* with a score of 2.02. 16 S rRNA sequencing was performed and the obtained sequence was compared with the NCBI 16 S and NCBI nucleotide databases and showed a 99–100% identity with *Helicobacter canis* (Fig. 2).

Antimicrobial susceptibility was performed using gradient E-test methods on BBA plates under micro-aerophilic conditions and incubated for 24 h. MICs are demonstrated in Fig. 1d.

*Clinical course* During the course of treatment, the erythema and swelling of the leg improved. Subsequently, the patient was discharged and treated with a combination of oral amoxicillin and doxycycline (once antimicrobial susceptibility results became available) for 14 days. The combination of amoxicillin and doxycycline was chosen because a case of recurrence with amoxicillin/clavulanic acid as single therapy was described [6].

Upon inquiry patient turned out to own a dog. She couldn’t remember being bitten or scratched by the dog.

*Patient 3*, a 69-year-old man with a history of deep venous thrombosis, male hypogonadism and hypospadias presented to the Emergency department with a two day history of fever, chills and blue discoloration of fingers and ears. Laboratory examination revealed an elevated C-reactive protein (86 mg/L) and a leukocytosis (10,2 × 10^9^/L). On the CT-scan on the day of admission, which was performed because of sepsis of unknown origin, an atrophic spleen was observed. Because of severe septic shock with multi-organ failure the patient was transferred to the intensive care unit and required hemodynamic support, mechanical ventilation and renal replacement therapy. Antibiotic treatment with ceftriaxone and tobramycin was started for suspected urosepsis.

**Fig. 3:**
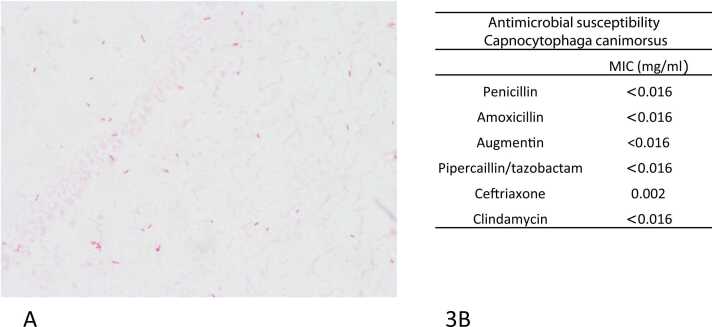
**a.** Gram stain of the blood culture bottle from patient 3 showing fusiform gram-negative rods **b.** Antimicrobial susceptribility results. MICs for penicillin, amoxicillin, amoxicillin/clavulanic acid, piperacillin/tazobactam, ceftriaxone and clindamycine.

*Microbiology* The day after admission, 4 blood cultures were reported positive (time to detection ranging from 13 h to 18 h). Gram stain revealed the presence of fusiform gram-negative rods (Fig. 3a). The blood cultures were sub-cultured onto BA, CHOC and BBA with 5% CO2 at 35Cº. MALDITOF MS identified *Capnocytophaga canimorsus* with a score of 2.25. Antimicrobial susceptibility was performed using gradient E-test methods on BBA plates and incubated in an atmosphere with 5% CO2. Because of slow growth antimicrobial susceptibility results were available after 14 days. MICs are demonstrated in Fig. 3b**.**

*Clinical course* Antibiotic treatment was switched to piperacillin/tazobactam 4500 mg 4 times daily while awaiting susceptibility results, because ß-lactamase production has been described in *Capnocytophaga* species although rare in *Capnocytophaga canimorsus* [7]. Six days after admission the patient could be extubated and was transferred to the internal medicine ward. Antibiotic treatment was continued for a total of 14 days. Renal replacement therapy could be discontinued 17 days after admission. After an admission of one month the patient was discharged to a physical rehabilitation center. As a consequence of the severe sepsis the patient developed dry necrosis of all fingers and toes. In a conservative approach there was a remarkable recovery. However, several auto-amputations and surgical amputations of distal phalanges of both hands and feet still occurred. Nine months later all defects were healed.

Upon inquiry, the patient turned out to be the owner of two dogs. One of the dogs probably licked a small laceration on the ear of the patient, which may have caused the infection.

## Discussion

Zoonotic diseases can be transmitted by infected saliva, aerosoles and/or infected urine or feces [1].

Here, we described three patients with severe pet-related zoonotic infections. In the second patient the route of transmission was unknown, although this patient lived in close contact with a dog. In the first and third patient the transmission was probably caused by licking of non-intact skin.

### Pasteurella multocida

*Pasteurella* are commensals of the oropharynx of healthy animals. *Pasteurella spp* are facultative anaerobic gram negative coccobacilli. *Pasteurella multocida* and *Pasteurella canis* are the most common species causing human disease. *Pasteurella multocida* grows well on blood, chocolate and Mueller-Hinton agar. Growth on MacConkey agar is uncommon [8]. Most human infections are caused by bites from dogs or cats or licking of non-intact skin [9], [10]. Infections can occur in healthy individuals, however immunocompromised patients, elderly and young children have a higher risk for developing severe disease. The patient we describe had a history of diabetes and peripheral vascular disease. Giordano et al. retrospectively studied 44 patients with Pasteurella infections. 6/25 (24%) patients with a skin infection following dog or cat bites had a history of diabetes or peripheral vascular disease. Besides diabetes, risk factors for severe disease were cirrhosis, COPD, malignancies or other conditions associated with an immunocompromised state [11]. Infections with *Pasteurella multocida* can be divided in three categories 1. Skin and soft tissue infections following dog or cat bites or scratches. 2. Respiratory infections usually in patients with chronic pulmonary disease. 3. Systemic infections in immunocompromised hosts [6]. *Pasteurella* spp are usually susceptible to ß-lactam antibiotics, fluoroquinolones and trimethoprim-sulfamethoxazole. Penicillin resistance has been described. [11], [12].

### Helicobacter canis

Infections with *Helicobacter canis* are rare. The majority of case reports describe immunocompromised patients with dog contact and *Helicobacter canis* bacteremia. In a few case reports, as in the above mentioned case, cellulitis was reported [6], [13], [14]. *Helicobacter canis*, a slightly curved gram-negative rod, can frequently be found in the gastrointestinal tract of cats and dogs, but also sheep [15], [16], [17], [18]. Dog bites or scratches prior to infection have not been described, therefore the most likely route of transmission is the fecal-oral route.

*Helicobacter canis* is a fastidious micro-organism and therefore, isolation can be difficult. In our laboratory, growth of *Helicobacter canis* was observed on BBA and BHM agar under micro-aerophilic conditions. A summary of the described case reports can be found in Table 1.

**Table 1:** Summary of the described Helicobacter canis case reports.

**Author**	**Patient**	**Clinical symptoms**	**Pet exposure**	**Culture**	**Antimicrobial susceptibility**	**Treatment**
Leemann 2006	Immunocompetent	Fever and cellulitis	Cat and dog	5% sheep blood agar and brucella agar with hemin, vit K1 and cysteine (anaerobic and microaerophilic)	E-test: amoxi 0,38; augm 0.094; ceftriaxone 0.75; piptazo 1; imipenem 0.047; metronidazole 0.064; clinda 0.094	Augmentin 10 days; recurrence ceftriaxone iv 2 weeks
Gerrard 2001	X-linked hypogammaglobulinemie	Recurrent fever episodes	Dog	sheep blood agar and chocolate agar aerobic and anaerobic	not performed	repeated courses AB, 5 weeks ceftriaxone; 1 week genta + ampicillin and 4 weeks cipro; because of recurrence 5 month doxy + metronidazole
Prag 2007	7-month-old child	Fever	Cat	5% sheep blood agar, brucella agar and chocolate agar aerobically, anaerobically and microaerophilic	Resistant to cephalotin (30 µm) sensitive to nalidixic acid	ampicillin and gentamicin iv followed by oral mecillinam for 10 days
Alon 2010	Gastric lymphoma	Fever	Dog	Culture positive?	not performed	Amoxicilline and clarithromycin 2 days; switch to piptazo because of general deconditioning 7 days followed by amoxicillin 4 weeks
van der Vusse 2013	Renal transplant	Fever	Dog	No growth	not performed	Cefuroxim 3 days and ciprofloxacin
Abidi 2013	Common variable immunodeficiency	Recurrent fever		chocolate agar microaerophilic	not performed	Meropenem 8 days, ceftriaxone iv 2 weeks and oral doxycycline 6 weeks
Shakir 2017	End-stage renal disease	Cellulitis	Dog	sheep blood agar microaerobically	not performed	Vanco 1x,7 days doxy and 8 weeks amoxicillin/clavulanic acid
This manuscript	Reumatoide artritis	Cellulitis	Dog	BBA and BHM microaerophilic		Augmentin iv 8 days, amoxicillin + doxycycline 2 weeks

In most of the case reports described growth was observed under micro-aerophilic conditions and therefore this seems the most appropriate culture condition. Antimicrobial susceptibility testing was performed on BBA agar. MALDITOF MS can identify *Helicobacter canis* which was confirmed by 16 S rRNA sequencing in our laboratory. The optimal treatment regimen for *Helicobacter canis* bacteremia is unknown. Relapses (or reinfection?) after initial treatment have been described in two out of 8 case report [6], [14].

### Capnocytophaga canimorsus

*Capnocytophaga canimorsus* is part of the normal oral flora of cats and dogs. *Capnocytophaga canimorsus* infection can lead to severe disease, with fulminant sepsis [19]. Immunocompromised patients are at greatest risk, particularly patients with asplenia. The fulminant course in the patient we describe, was attributed to functional asplenia with an atrofic spleen seen on CT imaging. A nationwide survey in The Netherlands in 2011 showed that infection with *Capnocytophaga canimorsus* is rare with an incidence rate of 0.67 cases per million population [20]. In most cases, patients report a history of a dog bite or scratch, although in some cases only living in close contact with dogs was reported [21]. The most severe presentation is acute sepsis, characterized by fever, hypotension, acute renal failure and disseminated purpura, which may progress to cutaneous gangrene. The case-fatality rate is approximately 25%. Other types of infections such as endocarditis, arthritis and meningitis have been described [22].

*Capnocytophaga canimorsus* is a Gram-negative, slow-growing, non-spore-forming facultative anaerobic rod [23]. The genus *Capnocytophaga*, comprises capnophilic species found in the oral cavities of humans and domestic animals [24]. In blood culture (aerobic or anaerobic flask) growth can be observed after 1–14 days (average 4–5 days). *Capnocytophaga spp* are facultative anaerobic and require enrichment with CO2 (5–10%) for optimum growth (capnophilic). The most optimal growth occurs at 35–37 °C on either blood or chocolate agar. No growth is observed on MacConkey agar. MALDI-TOF MS can accurately identify *Capnocytophaga canimorsus* using an enriched database [25].

*Capnocytophaga canimorsus* is usual susceptible to betalactam antibiotics, however *Capnocytophaga species* have been described to produce beta-lactamases [7]. Therefore, antimicrobial susceptibility testing should be performed if possible.

*Pasteurella multocida*, *Helicobacter canis* and *Capnocytophaga canimorsus* are normal commensals of the oral cavity in dogs and cats. The above described infections are rare but severe infections and the risk of contracting a dog-related zoonotic infection is higher in immunocompromised patients. Especially patient 1 and 3 still suffer from the consequences of this severe infections. Early recognition and treatment can reduce morbidity and mortality. A complete medical history can be helpful for early diagnosis of these difficult to culture micro-organisms.

With this case report we would like to emphasize the importance of awareness for pet-related zoonotic infections in both pet owners and doctors, which can lead to early recognition, diagnosis and treatment.

All authors declare that they have no conflicts of interest. We received no specific grant from any funding agency.

Written informed consent was obtained.

## CRediT authorship contribution statement

Please specify the contribution of each author to the paper, e.g. study design, data collections, data analysis, writing, others, who have contributed in other ways should be listed as contributors. E.M. van Wezel, N.E.L. Meessen and M. Wouthuyzen-Bakker: study design, data collection,writing. E.S.J. van der Beek, M.A.N. Siebrecht, A.J. Stel: data collection and writing.
